# Long-term cardiovascular outcomes of biodegradable polymer drug eluting stents in patients with diabetes versus non-diabetes mellitus: a meta-analysis

**DOI:** 10.1186/s12933-023-01962-w

**Published:** 2023-08-29

**Authors:** Hong Wang, Quannan Zu, Hairong Tang, Ming Lu, Rongfa Chen, Zhiren Yang

**Affiliations:** 1 Department of Cardiology, The People’s Hospital of Guangxi Zhuang Autonomous Region, 530021 Nanning, Guangxi P. R. China; 2 College of Management and Economics, Tianjin University, 300072 Tianjin, P. R. China; 3 The State Key Laboratory Management and Control for Complex Systems, Institute of Automation, Chinese Academy of Sciences, 100190 Beijing, P. R. China

**Keywords:** Biodegradable polymer drug eluting stents, Durable polymer drug eluting stents, Percutaneous coronary intervention, Diabetes mellitus, Cardiovascular diseases, Stent thrombosis, Revascularization

## Abstract

**Background:**

Today, diabetes mellitus (DM) has become a worldwide concern. DM is a major risk factor for the development of cardiovascular diseases (CVD). Eligible patients with CVD are treated invasively by percutaneous coronary intervention (PCI) whereby a stent is implanted inside the coronary vessel with the particular lesion to allow sufficient blood flow. Newer scientific research have shown that even though associated with a lower rate of re-stenosis, first-generation drug eluting stents (DES) were associated with a higher rate of late stent thrombosis. Recently, newer stents, namely biodegradable polymer DES (BP-DES) have been developed to overcome the safety issues of earlier generation DES. In this analysis we aimed to systematically compare the long term (≥ 12 months) adverse cardiovascular outcomes observed in DM versus non-DM patients who were implanted with BP-DES.

**Methods:**

Cochrane central, MEDLINE (Subset PubMed), EMBASE, Web of Science, http://www.ClinicalTrials.gov and Google scholar were searched for relevant publications involving BP-DES in patients with DM versus non-DM and their associated adverse cardiovascular outcomes. The mean follow-up time period ranged from 12 to 120 months. Data analysis was carried out with the latest version of the RevMan software (version 5.4). Based on the Mantel-Haenszel test, risk ratios (RR) with 95% confidence intervals (CI) were calculated and used to represent the results following analysis.

**Results:**

Seven (7) studies with a total number of 10,246 participants were included in this analysis. Stents which were implanted during PCI were BP-DES. Participants were enrolled from the year 2006 to 2013. Our current results showed that in patients who were implanted with BP-DES, the risks of major adverse cardiac events (RR: 1.30, 95% CI: 1.18–1.43; P = 0.00001), myocardial infarction (RR: 1.48, 95% CI: 1.14–1.93; P = 0.003), all-cause mortality (RR: 1.70, 95% CI: 1.29–2.23; P = 0.0002), cardiac death (RR: 1.93, 95% CI: 1.28–2.93; P = 0.002), target vessel revascularization (RR: 1.35, 95% CI: 1.03–1.77; P = 0.03), target lesion revascularization (RR: 1.28, 95% CI: 1.07–1.54; P = 0.007) and target lesion failure (RR: 1.79, 95% CI: 1.52–2.12; P = 0.00001) were significantly higher in the DM group. Definite and probable stent thrombosis (RR: 1.80, 95% CI: 1.28–2.55; P = 0.0009) were also significantly higher in the DM group.

**Conclusions:**

Diabetes mellitus was an independent risk factor associated with long term adverse cardiovascular outcomes following PCI with BP-DES.

**Supplementary Information:**

The online version contains supplementary material available at 10.1186/s12933-023-01962-w.

## Background

Today, diabetes mellitus (DM) has become a worldwide concern [1]. DM is a major risk factor for the development of cardiovascular diseases (CVD). The global burden of CVD is disproportionately borne by patients with DM [2]. Several mechanisms could explain the effects of DM on the cardiovascular system [3]. High blood sugar levels contribute to oxidative stress through the production of mitochondrial superoxide, NADPH reduction through the accumulation of polyol, and the synthesis of AGE through the non-enzymatic oxidation of glycoproteins, all of which could cause endothelial damage within coronary arteries giving rise to CVD. DM is also associated with high platelet reactivity which might result in acute coronary syndrome (ACS), further complicating CVD [4].

Patients with CVD are often treated invasively by percutaneous coronary intervention (PCI) whereby a stent is implanted inside the coronary vessel with the particular lesion to allow sufficient blood flow to the heart muscles [5]. Several types of coronary stents have been developed [6]. Bare metal stents (BMS) were the first stents which were used during PCI [7]. However, due to the higher risk of re-stenosis and early stent thrombosis with BMS, a drug eluting stent (DES) was developed [8]. Even though patients with DM often have more complicated lesions and more severe disease, including more extensive and diffuse atherosclerosis, with increased risk of triple vessel diseases, and left main coronary occlusions, DES was a good option when the patients were candidates for PCI [9].

Unfortunately, with recent progress in medicine and technology, newer scientific research have shown that even though associated with a lower rate of re-stenosis, first-generation DES were associated with a higher rate of late stent thrombosis [10]. Explanations which were given were based on the fact that the permanent polymers of the DES which contained the anti-proliferative drug partially contributed to the delayed vascular healing, which might be a potential cause for the late stent thrombosis in patients who were revascularized with first generation DES [11]. In addition, DM was associated with platelet dysfunction [12], and antiplatelet hyporesponsiveness [13] which could further increase the risk of stent thrombosis in such patients. Therefore, in CVD patients with DM, the development of a more effective and safer DES could potentially decrease adverse outcomes following PCI.

Recently, newer stents, namely biodegradable polymer DES (BP-DES) were developed to overcome the safety issues of earlier generation DES [14]. The BP-DES system incorporates a biodegradable polymer carrier containing the anti-proliferative drug which is only applied on the luminal surface of the stent platform, thus, limiting its exposure to blood and therefore, the abluminal coating might enhance the attachment of endothelial progenitor cells from the peripheral circulation, or in-growth of the endothelial tissue from the proximal and the distal edges of the stents [15]. Thus, a major advantage of this BP-DES could be the fact that it could be associated with similar long term outcomes as the first generation DES, but with a more favorable safety profile based on a reduction of late stent thrombosis in patients with DM. However, this benefit has still not been confirmed [16].

All around the globe, DM is rising at an alarming rate, resulting in an increase in cardiovascular complications and deaths. Therefore, scientists and medical professionals should focus on further clinical research to provide the best treatment and management of patients with DM. The most recent BP-DES have seldom systematically been compared in patients with DM versus patients without DM.

In this analysis we aimed to systematically compare the long term (≥ 12 months) adverse cardiovascular outcomes observed in DM versus non-DM patients with CVD who were implanted with BP-DES.

## Methods

### Search databases

Cochrane central, MEDLINE (Subset PubMed), EMBASE, Web of Science, http://www.ClinicalTrials.gov and Google scholar were searched for relevant publications involving BP-DES in patients with DM versus non-DM.

### Search strategies

The above mentioned databases were searched for relevant publications using the following search terms: ‘biodegradable polymer drug eluting stents and diabetes mellitus’, ‘biodegradable polymer DES and diabetes mellitus’, ‘biodegradable polymer drug eluting stents and DM’.

After going through the relevant publications, their reference lists were also searched for suitable publications.

This meta-analysis was performed in accordance with the Preferred Reporting Items in Systematic reviews and Meta-Analyses (PRISMA) guideline.

### Inclusion and exclusion criteria

The following inclusion and exclusion criteria were considered:

- Inclusion criteria:

- Studies comparing biodegradable polymer DES in patients with DM and non-DM;
- Reported adverse cardiovascular outcomes as the endpoints;
- English publications.

(b) Exclusion criteria:

- Systematic reviews, meta-analyses and literature reviews;
- Studies which were not based on patients with DM;
- Studies that did not include a comparison of biodegradable polymer DES between DM and non-DM;
- Studies that did not report adverse cardiovascular outcomes as the endpoints;
- Duplicated studies or studies which were based on the same trial.

### Outcomes and the duration of follow-up time period

The outcomes which were reported in the original studies have been listed in Table 1. The follow up time period of each studies has been listed in the same table.

**Table 1:** Outcomes reported and duration of follow-up time period

Study	Outcomes reported in the original studies	Duration of follow-up time period (months)
**Iglesias2019** [17]	All-cause mortality, cardiac death, MI, repeated revascularization, TLR, TVR, cerebrovascular events, TIA, stroke, ischemic stroke, intra-cerebral hemorrhagic stroke, TLF, TVF, MACEs, definite ST, definite or probable ST	60 months
**Lee2017** [18]	TLF	24 months
**Lenz2021 [19]**	MACEs, all-cause mortality, cardiac death, MI, TLR, definite ST, probable ST, definite/probable ST	120 months
**Li2012** [20]	All-cause mortality, cardiac death, MI, TLR, MACEs, repeated revascularization	48 months
**Rola2021** [21]	MACEs, all-cause mortality, cardiac death, MI, ST, repeated revascularization, TVR, TLR	12 months
**Tang2018** [22]	MACEs, all-cause mortality, cardiac death, MI, ST, repeated revascularization, TVR, TLR, stroke	24 months
**Wiermer2017** [23]	Cardiac death, all-cause mortality, MI, TLR, TVR, MACEs, TLF, definite and probable ST	60 months

Abbreviations: TLF: Target lesion failure; TLR: Target lesion revascularization, MI: Myocardial infarction, ST: Stent thrombosis; TVR: Target vessel revascularization, TIA: Transient ischemic attack, TVF: Target vessel failure, MACEs: Major adverse cardiac events

The endpoints which have been assessed in this analysis were:

- Major adverse cardiac events (MACEs) consisting of a combination of all-cause mortality, myocardial infarction and revascularization;
- All-cause mortality;
- Cardiac death;
- Myocardial infarction (MI);
- Definite and probable stent thrombosis;
- Target vessel revascularization (TVR);
- Target lesion revascularization (TLR);
- Target lesion failure (TLF).

The duration of the follow-up time period ranged from 12 to 120 months (1–10 years).

### Data extraction and quality assessment

The authors independently extracted data including the time period of participants’ enrollment, the type of study, the outcomes which were reported, the total number of events associated with each outcome, the type of biodegradable polymer, the total number of patients with DM and non-DM, the baseline features including the mean age, percentage of male patients, percentage of participants with hypertension, dyslipidemia and current smoker, the cardiovascular and antiplatelet drugs which were used as well as the angiographic features of the lesions including the percentage of patients with lesions in the left main coronary artery, left anterior descending artery, left circumflex and right coronary artery.

Any disagreement which occurred during this data extraction process was carefully discussed among the authors and if a decision could not be reached, the corresponding author was contacted and he was the one to make a final decision.

The methodological quality of the trials and observational studies was assessed by the recommendations of the Cochrane collaboration [24] and the Newcastle Ottawa Scale (NOS) [25] respectively.

Grades were allotted to denote a low, moderate or high risk of bias for the randomized trials, whereas a ‘star system’ was used to represent bias risk assessment for the observational studies (Table 2). This assessment of observational studies was based on the design of the respective study, content and ease of use directed to the task of incorporating the quality assessments on three broad perspectives: the selection of the study groups; the comparability of the groups; and the ascertainment of either the exposure or outcome of interest for case-control or cohort studies respectively. The higher the number of stars, the better the methodological quality of the study.

**Table 2:** The methodological quality assessment of the observational studies

For the observational studies	Lee2017	Rola2021	Tang2018	Wiermer2017
*Selection*				
Representative of the exposed cohort	*	*	*	*
Selection of the external control	*	*	*	*
Ascertainment of exposure	x	x	x	x
Outcome of interest not present at the start of the study	*	*	*	*
***Comparability***				
Main factor and additional factor based on comparability of cohorts	*	*	*	*
***Outcome***				
Assessment of outcomes	*	*	*	*
Sufficient follow up time	*	*	*	*
Adequacy of follow up	*	*	*	*

Abbreviations: √ (present); x (absent or not reported)

### Statistical analysis

This is a meta-analysis, and the data analysis was carried out with the latest version of the RevMan software (version 5.4).

Studies which have been included in this analysis will differ from each other. Any kind of variability among studies in a meta-analysis is termed heterogeneity.

Two simple statistic test were used to assess heterogeneity among the subgroups: (1) the Q statistic test whereby a P value less or equal to 0.05 associated with a particular subgroup was considered statistically significant, (2) the I^2^ statistic test whereby heterogeneity decreased with a decreased I^2^ value. For a subset having an I^2^ value less than 50%, a fixed effect statistical model was used, whereas for a subset having an I^2^ value above 50%, a random effect statistical model was used.

Based on the Mantel-Haenszel test, risk ratios (RR) with 95% confidence intervals (CI) were calculated and used to represent the results following analysis.

Sensitivity analysis was also carried out. Sensitivity analysis was conducted to countercheck whether the final result was influenced by one particular study. This sensitivity analysis was carried out by an exclusion method whereby among all the studies which were involved with one particular outcome, each study was excluded one by one, and a new analysis was carried out each time and compared with the main analytical result.

Publication bias was also assessed through funnel plots.

### Ethical approval

An ethical approval or board review approval was not required for this study since data were obtained from previously published original studies, and no experiment on animals or humans was carried out by any of the authors.

## Results

### Search outcomes

The PRISMA guideline was followed in this systematic review and meta-analysis [26]. A total number of 235 publications were obtained through search databases. The authors carefully assessed the titles and abstracts, and therefore an initial elimination of studies was carried out prior to assessing the full text articles.

Therefore, following this initial elimination whereby 179 publications were eliminated, we finally short-listed 56 full text articles which we assessed for eligibility. Further elimination were carried out based on the following:

- Studies that did not involve non-DM group as the control group (n = 13);
- Studies which reported platelet reactivity instead of adverse cardiovascular outcomes (n = 3);
- Case studies, editorials and erratum (n = 5);
- Duplicated studies or studies which were based on the same trial (n = 28).

**Fig. 1:**
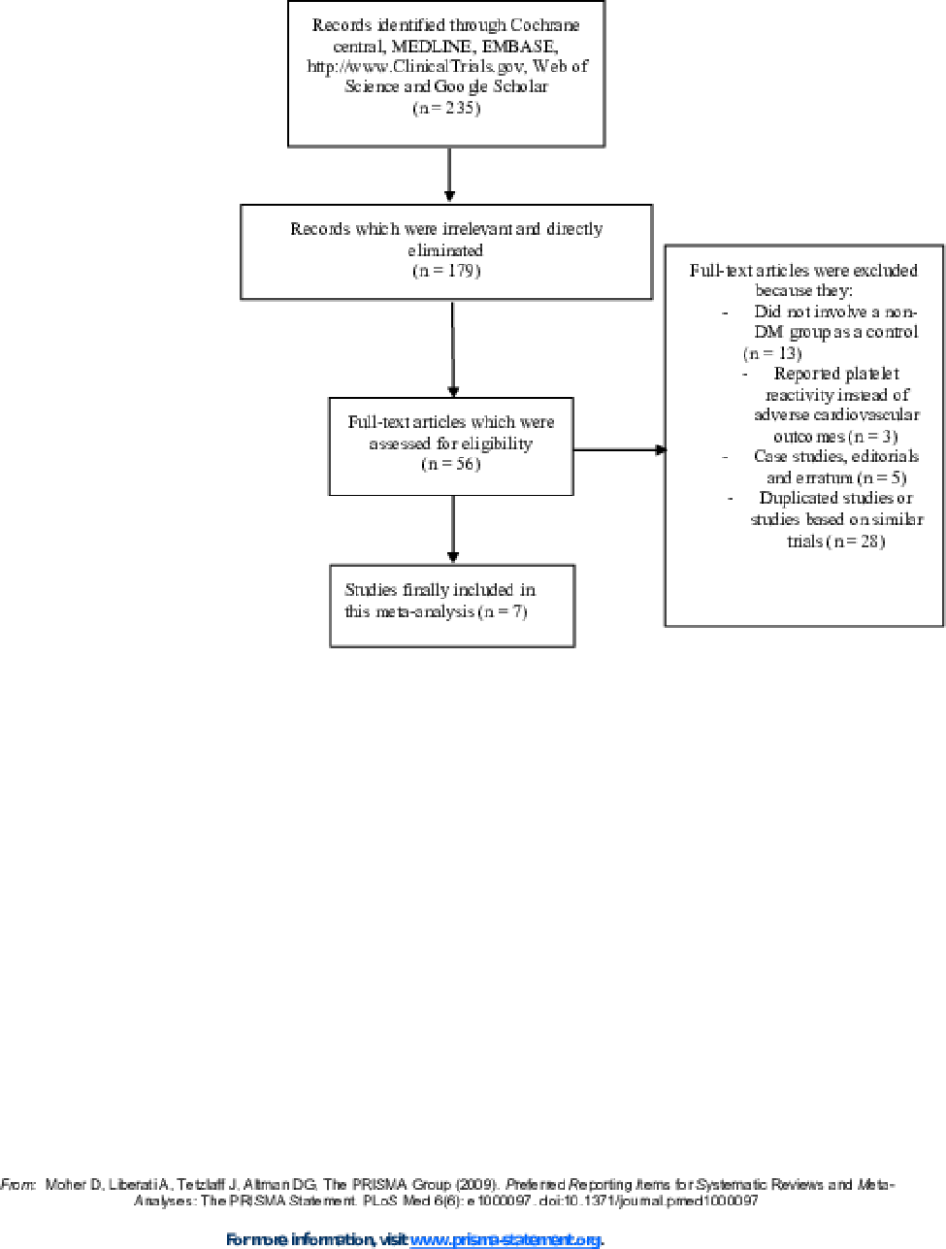
Flow diagram showing the study selection

Finally, only 7 studies [17–23] were included in this meta-analysis. The flow diagram of the study selection has been illustrated in Fig. 1.

### General and baseline features of the studies

Seven (7) studies with a total number of 10,246 participants were included in this analysis whereby 2747 participants were DM patients and 7499 participants were non-DM as shown in Table 3. The stents which were implanted during PCI were BP-DES. Participants were enrolled from the year 2006 to 2013.

**Table 3:** General features of the studies which were included in this analysis

Studies	No of DM participants (n)	No of non-DM participants (n)	Type of stents	Type of study	Period of enrollment (years)
**Iglesias2019**	257	806	BP-SES	Trial	2012–2013
**Lee2017**	306	693	BP-BES	Observational	2010–2012
**Lenz2021**	376	923	BP-SES	Trial	2007–2008
**Li2012**	440	1637	BP-SES	Trial	2006
**Rola2021**	59	110	BP-DES	Observational	2015–2020
**Tang2018**	421	1151	BP-DES	Observational	2013
**Wiermer2017**	888	2179	BP-DES	Observational	2008–2009

Abbreviations: DM: Diabetes mellitus; BP-SES: Biodegradable polymer sirolimus eluting stents; BP-BES: Biodegradable polymer biolimus eluting stents; BP-DES: Biodegradable polymer drug eluting stents

The baseline features of the participants were listed in Table 4. The mean age of the participants with DM varied from 59.7 to 68.6 years, whereas the mean age of the non-DM participants ranged from 58.5 to 66.5 years. The mean percentage of male participants ranged from 69.6 to 80.3% as shown in Table 4. The percentage of DM and non-DM participants with hypertension, dyslipidemia and smokers were also listed in Table 4.

**Table 4:** Baseline features of the studies which were included in this analysis

Studies	Mean age (years)	Males (%)	HBP (%)	DL (%)	Smoker (%)
Features	DM/NDM	DM/NDM	DM/NDM	DM/NDM	DM/NDM
**Iglesias2019**	68.6/65.3	77.0/76.9	86.0/63.0	73.5/65.0	21.8/31.4
**Lee2017**	64.2/64.2	69.6/69.6	60.5/60.5	37.7/37.7	-
**Lenz2021**	66.9/66.5	72.9/76.3	78.5/65.2	68.4/66.2	13.3/16.5
**Li2012**	62.3/60.1	64.8/75.9	68.9/52.1	26.7/31.0	-
**Rola2021**	66.1/64.8	-	98.3/90.1	79.6/75.5	-
**Tang2018**	59.7/58.5	74.6/75.8	74.6/63.1	72.7/62.3	14.3/10.3
**Wiermer2017**	66.4/63.5	72.3/80.3	81.3/64.0	76.8/68.6	17.8/28.7

Abbreviations: DM: Diabetes mellitus; NDM: Non-diabetes mellitus; HBP: High blood pressure; DL: Dyslipidemia

Table 5 lists the antiplatelet medications as well as the other cardiac drugs which are used by the participants. All the participants were on dual antiplatelet therapy including aspirin and a P2Y12 inhibitor (clopidogrel or prasugrel or ticagrelor) as shown in Table 5. Other medications included angiotensin receptor blockers, angiotensin converting enzyme inhibitors, statins, beta-blockers, calcium channel blockers as the main drugs.

**Table 5:** The antiplatelet agents and the other cardiac drugs used by the participants

Studies	Anti-platelets which were used	Other cardiac medications
**Iglesias2019**	ASA plus clopidogrel or prasugrel or ticagrelor (DAPT)	Statin, ACEI or ARB, beta-blocker, vitamin K oral anticoagulant or non-vitamin K oral antagonist
**Lee2017**	DAPT with ASA and any P2Y12 inhibitor	-
**Lenz2021**	DAPT	-
**Li2012**	DAPT	-
**Rola2021**	ASA plus clopidogrel or prasugrel or ticagrelor (DAPT)	Statin, ACEI or ARB, beta-blocker
**Tang2018**	ASA plus clopidogrel	Statin, CCB, beta-blocker, nitrates
**Wiermer2017**	DAPT	-

Abbreviations: DAPT: Dual antiplatelet therapy; ASA: Aspirin; CCB: Calcium channel blocker; ACEI: Angiotensin converting enzyme inhibitor; ARB: Angiotensin receptor blocker

Table 6 lists the angiographic features including the location of the coronary lesions, the number of treated lesions per patients, and the number of stents implanted per lesion.

**Table 6:** The angiographic features reported in each study

Study	Iglesias2019	Lee2017	Lenz2021	Li2012	Rola2021	Tang2018	Wiermer2017
Participants	DM/NDM	DM/NDM	DM/NDM	DM/NDM	DM/NDM	DM/NDM	DM/NDM
*Target vessel location per lesion (%)*							
**Left main coronary artery involved**	2.50/1.60	2.10/2.10	-	-	-	2.10/1.30	-
**Left anterior descending artery involved**	35.6/42.4	54.1/54.1	42.4/45.2	-	35.5/40.0	89.5/91.2	-
**Left circumflex artery involved**	24.2/22.9	28.7/28.7	29.4/25.7	-	32.2/25.4	13.3/14.2	-
**Right coronary artery involved**	32.1/31.6	33.4/33.4	28.2/29.1	-	30.5/34.5	22.3/15.9	-
***No of treated lesions per patient (n)***							
**1**	155/528	534/534	47/223	-	-	70/340	-
**2**	73/193	306/306	143/395	-	-	143/379	-
**3**	22/62	159/159	370/772	-	-	185/378	-
**4 or more**	7/23	-	-	-	-	-	-
**No of stents per lesion (mean with SD)**	1.32 ± 0.64/1.30 ± 0.60	1.30 ± 0.70/1.30 ± 0.70	-	1.92 ± 1.20/1.92 ± 1.20	-	1.70 ± 0.90/1.67 ± 0.88	1.18 ± 0.58/1.20 ± 0.57

Abbreviations: DM: Diabetes mellitus; NDM: Non-diabetes mellitus; SD: Standard deviation

### Main results of this analysis

**Fig. 2:**
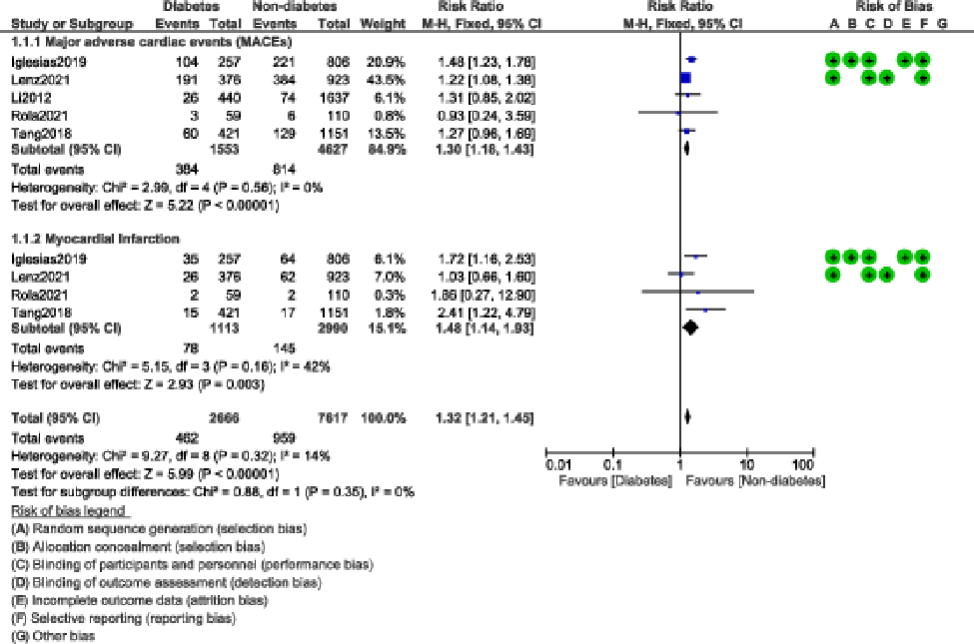
Major adverse cardiac events and myocardial infarction observed with BP-DES in patients with versus without DM

**Fig. 3:**
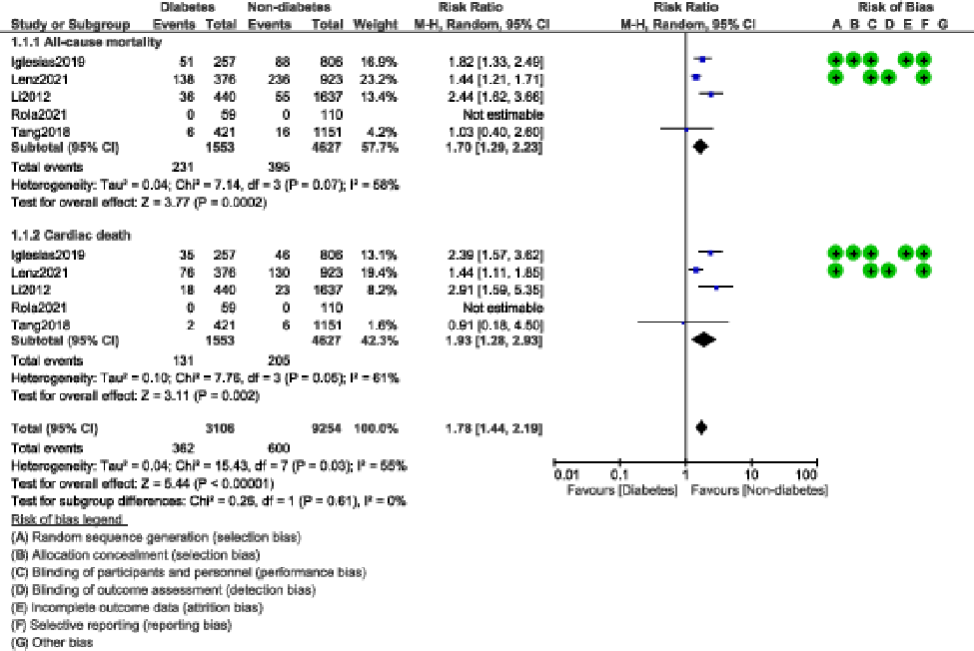
Cardiovascular outcomes observed with BP-DES in patients with versus without DM

During a mean follow-up time period ranging from one to ten years, our current results showed that in patients who were implanted with BP-DES, the risks of MACEs and MI were significantly higher in patients with DM with RR: 1.30, 95% CI: 1.18–1.43; P = 0.00001; I^2^ = 0% and RR: 1.48, 95% CI: 1.14–1.93; P = 0.003; I^2^ = 42% when compared to non-DM participants as shown in Fig. 2. All-cause mortality (RR: 1.70, 95% CI: 1.29–2.23; P = 0.0002); I^2^ = 58%, and cardiac death (RR: 1.93, 95% CI: 1.28–2.93; P = 0.002); I^2^ = 61% were also significantly higher in the DM group as shown in Fig. 3.

**Fig. 4:**
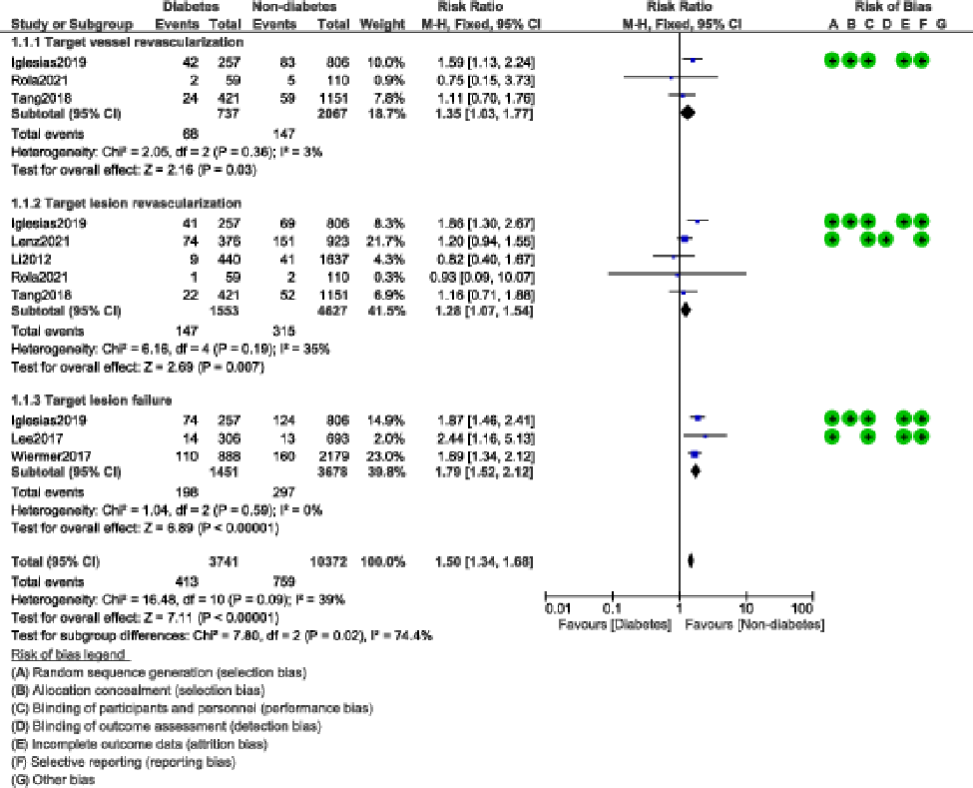
Repeated revascularization observed with BP-DES in patients with versus without DM

The risk of TVR (RR: 1.35, 95% CI: 1.03–1.77; P = 0.03); I^2^ = 3%, TLR (RR: 1.28, 95% CI: 1.07–1.54; P = 0.007); I^2^ = 35% and TLF (RR: 1.79, 95% CI: 1.52–2.12; P = 0.00001); I^2^ = 0% were also significantly higher in the DM group as shown in Fig. 4.

**Fig. 5:**
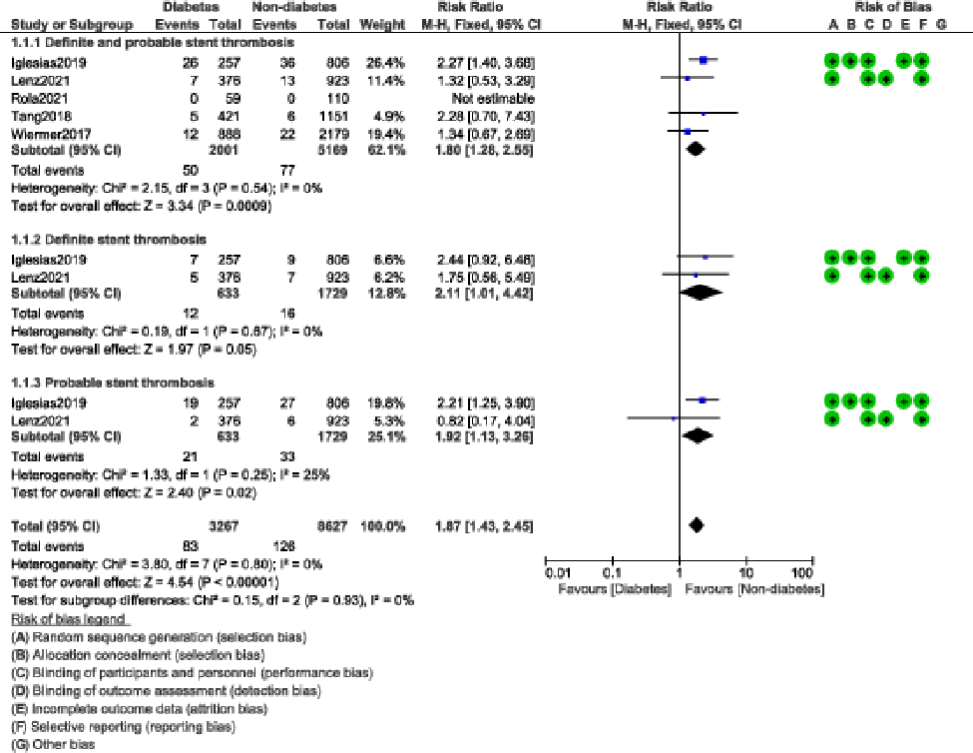
Stent thrombosis observed with BP-DES in patients with versus without DM

Definite and probable stent thrombosis (RR: 1.80, 95% CI: 1.28–2.55; P = 0.0009); I^2^ = 0%, specifically definite stent thrombosis (RR: 2.11, 95% CI: 1.01–4.42; P = 0.05); I^2^ = 0% and probable stent thrombosis (RR: 1.92, 95% CI: 1.13–3.26; P = 0.02); I^2^ = 25% were also significantly higher in the DM group as shown in Fig. 5.

**Fig. 6:**
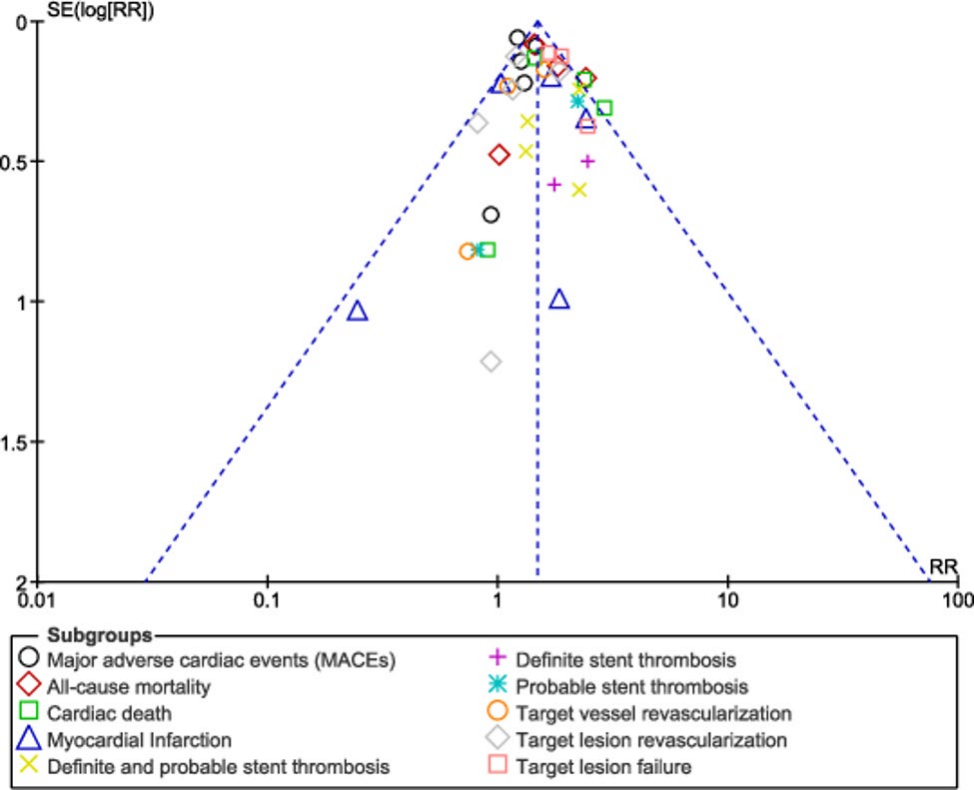
Funnel plot showing publication bias (**A**)

**Fig. 7:**
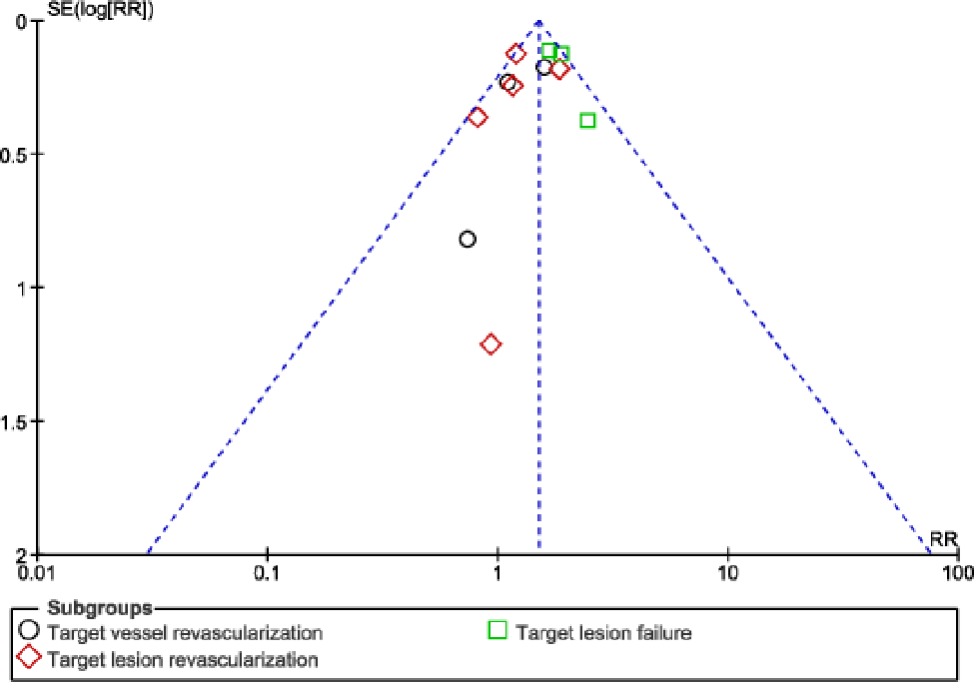
Funnel plot showing publication bias (**B**)

**Fig. 8:**
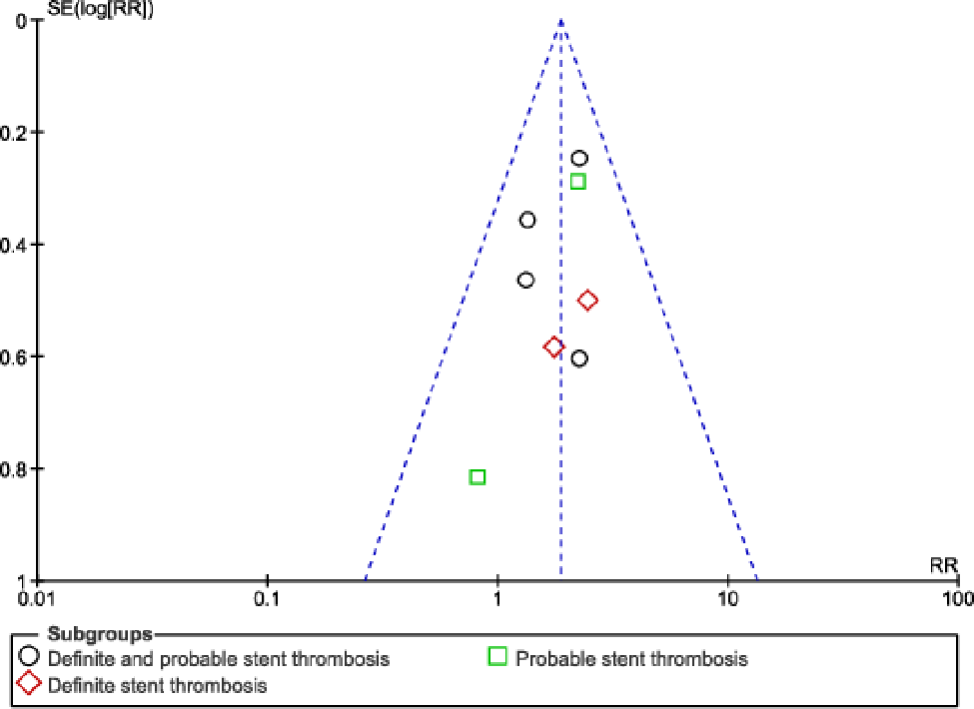
Funnel plot showing publication bias (**C**)

No significant difference in result was observed during sensitivity analysis, implying that the results of this analysis were not influenced by any particular study. Publication bias was visually observed through the funnel plots. Based on this assessment, there was little evidence of publication bias among the studies that were included in this analysis as shown in Figs. 6, 7 and 8.

## Discussion

DM patients with CVD are at greater risk of long term stent thrombosis following PCI with first generation DES [28]. Considering the fact that the polymer in DES could be a source of stent thrombosis in these patients with DM [29], the new BP-DES were developed. In this analysis, we aimed to systematically assess the cardiovascular outcomes in DM versus non-DM patients who were implanted with BP-DES.

The results of this analysis showed that MACEs, MI, all-cause mortality, repeated revascularization, TLF, and stent thrombosis were all significantly higher in the DM group compared to the DM group. The studies which were included in this analysis had a duration of follow up time period ranging from 12 months to 120 months.

Our current analysis showed that clinical outcomes including stent thrombosis continued to be worse in patients with DM despite of undergoing PCI with newer BP-DES whereby the polymer which might have been one of the causes of stent thrombosis, was eliminated with time. In addition, all patients were on dual antiplatelet drugs following stent implantation. Therefore, it could be possible that other potential factors in these patients with DM, including antiplatelet hyporesponsiveness and platelet hyperactivity and endothelial dysfunction could be more influential and have a greater impact compared to the polymer in DES to cause late stent thrombosis.

Scientific reports have shown diabetic status to affect the short and long term outcomes following PCI with any type of stents, through several pathophysiological mechanisms including the acceleration of atherosclerosis and promotion of endothelial dysfunction by hyperglycemia and insulin resistance. In addition, impaired vasodilation, exaggerated neointimal hyperplasia and platelet hyperactivity were observed in DM patients in comparison to non-DM patients, and these could have contributed to significantly higher adverse cardiovascular outcomes in DM patients compared to non-DM patients who were implanted with BP-DES. Furthermore, the proinflammatory environment of DM enhances the vasculoproliferative cascade in response to stent-mediated arterial injury and therefore, as a consequence, patients with DM experience both a higher recurrence of ischemic events related to new atherosclerotic lesions and worse cardiovascular outcomes for stent related complications including re-stenosis and stent thrombosis. Even though BP-DES have significantly reduced cardiovascular outcomes including late stent thrombosis in patients following PCI, our analysis showed that when DM and non-DM were compared following implantation with BP-DES, a significantly higher risk of adverse cardiovascular outcomes was still associated with DM patients. Could newer potent antiplatelet agents improve stent thrombosis in these patients with DM?

In a pre-specified subgroup analysis including participants from Germany, the authors demonstrated that during a follow-up time period of 10 years, BP-DES were associated with less stent thrombosis compared to durable polymer everolimus eluting stents in patients with DM and non-DM [19]. In patients with DM who were implanted with BP-DES, the percentage of stent thrombosis was 2.2% whereas in those DM patients who were implanted with durable polymer everolimus eluting stents, the percentage of stent thrombosis was 2.7%. But when stent thrombosis was compared in DM and non-DM participants who were implanted with BP-DES, stent thrombosis was 2.2% in DM population versus 1.6% in non-DM showing that DM participants were still at higher risk of stent thrombosis compared to non-DM participants whatever be the type of stents. A meta-analysis of randomized trial comparing BP-DES versus contemporary durable polymer DES in patients with DM, the authors showed that overall, BP-DES had similar efficacy and safety profiles in comparison to contemporary durable polymer DES in patients with DM [29]. The difference was observed when patients with DM were compared with non-DM patients.

In the ISAR-Test 5 trial whereby the authors aimed to show 10 year clinical outcomes of polymer free versus durable polymer new generation DES in patients with coronary artery disease with and without DM [30], 3002 participants were randomly assigned to polymer free sirolimus eluting stents or durable polymer zotarolimus eluting stents. Results of their study showed that at 10 years, both new generation DES showed comparable outcomes irrespective of diabetic status or polymer strategy. However, events rate after PCI in patients with DM were considerably higher when compared to patients without DM.

It is still not clear whether more potent antiplatelet agents could partially resolve this issue by decreasing stent thrombosis in these patients with DM [31]. When DES were upgraded to BP-DES and this new stent was compared in patients with DM and non-DM, the former were still at higher risk of adverse cardiovascular outcomes including stent thrombosis. It would now be the turn of antiplatelet agents to play the card. More potent antiplatelet agents could help to better manage such patients and reduce the rate of stent thrombosis [32]. A recent publication was based on the treatment in this new era showing the long term ticagrelor monotherapy use for the treatment of patients with DM following PCI [33]. The authors concluded that long term ticagrelor monotherapy after a short course of dual antiplatelet therapy was better, without significantly increasing bleeding risk in those patients with DM.

At last, several meta-analysis have compared BP-DES with durable polymer DES [34, 35], however, this is the first meta-analysis till date to compare the cardiovascular outcomes in DM versus non-DM participants who were treated with BP-DES.

This study has several limitations. First of all, even though all BP-DES were used, there were minor variations with those BP-DES which could have had an influence on the results. Another limitation could be the fact that other medications including antiplatelet drugs, which were ignored in this analysis, could have had an impact on the results. Moreover, the intensity of coronary artery disease was not similar in all the patients, and this could have had an impact on the outcomes too. Furthermore, even though the follow up time period varied from 12 months to 120 months, each study reported different follow up time periods. The angiographic features were also different for each patient and this could also have had an impact on the final result.

## Conclusion

Diabetes mellitus was an independent risk factor associated with long term adverse cardiovascular outcomes following PCI with BP-DES.

### Electronic supplementary material

Below is the link to the electronic supplementary material.

Supplementary Material 1

## Data Availability

All data and materials used in this research are freely available in electronic databases (Cochrane central, MEDLINE (Subset PubMed), EMBASE, Web of Science, http://www.ClinicalTrials.gov and Google scholar). References have been provided.

## Abbreviations

BP DES: Biodegradable polymer drug eluting stents
DES: Drug eluting stents
DM: Diabetes mellitus
NDM: Non-diabetes mellitus
RR: Risk ratios
PCI: Percutaneous coronary intervention
CVD: Cardiovascular diseases
